# Outbreak of Human Metapneumovirus Infection in Zoo, Slovenia

**DOI:** 10.3201/eid2608.200125

**Published:** 2020-08

**Authors:** Tina Uršič, Nika Lalek, Pavel Kvapil, Marjan Kastelic, Vasilij Cociancich, Irena G. Košnik, Miroslav Petrovec

**Affiliations:** University of Ljubljana, Ljubljana, Slovenia (T. Uršič, V. Cociancich, M. Petrovec);; Golnik University Clinic of Pulmonary and Allergic Diseases, Golnik, Slovenia (N. Lalek);; Ljubljana Zoo, Ljubljana (P. Kvapil, M. Kastelic);; National Institute of Public Health, National Laboratory of Health, Environment, and Food, Maribor, Slovenia (I.G. Košnik)

**Keywords:** metapneumovirus, viruses, infection, bronchopneumonia, outbreak, humans, chimpanzees, keeper, zoo, respiratory infections, Slovenia

## Abstract

We report a case of human metapneumovirus infection that spread from humans to chimpanzees and back to humans. Bronchopneumonia developed in 4 of 6 members of a chimpanzee family, and 2 subsequently died. The chimpanzees’ keeper also became ill. Sequencing showed 100% identity between virus sequences from chimpanzees and the keeper.

Apes are the closest nonhuman primate relative of humans and are therefore susceptible to many human pathogens. Chimpanzees have been kept at the Ljubljana Zoo in Slovenia since 1974. The zoo had never experienced a severe or fatal case of viral respiratory infection among the chimpanzee family, which consisted of 6 members (10, 11, 13, 15, 25, and 38 years of age). We report an outbreak of human metapneumovirus (hMPV) infection in chimpanzees and a zookeeper at this zoo.

This study was performed in accordance with the Helsinki Declaration. Written consent was obtained from the human patient and archived.

On June 19, 2013, four of the youngest chimpanzees at the zoo started showing signs of a cold (snorting, sneezing, and coughing). Veterinarians suspected a viral infection, but because of the possibility of secondary bacterial infection, the chimpanzees were given amoxicillin and clavulanic acid. The next day, clinical signs of pneumonia (apathy, dyspnea, and loss of appetite) appeared. The youngest chimpanzee died of respiratory failure on June 22; necropsy showed acute bronchopneumonia.

We tested animals for influenza A and B viruses, respiratory syncytial virus, hMPV, human coronaviruses (NL63, OC43, HKU1, and 229E), human bocavirus 1, human rhinoviruses, adenoviruses, and enteroviruses by using real-time reverse transcription PCR. Only hMPV was detected in nasal and throat swab specimens and lung tissue (*1*). The same day, the health of the other chimpanzees deteriorated.

The second-youngest chimpanzee that had signs of acute respiratory distress was sedated, ventilated, and given Ringer solution, bronchodilators, and intravenous antimicrobial drugs. However, it died on June 24 because of bronchopneumonia and pleural effusion, which was confirmed by necropsy.

In nasal and throat swab specimens and lung tissue, only hMPV and *Klebsiella pneumoniae* were detected. Histopathologic examination of hematoxylin and eosin–stained lung tissue samples of both chimpanzees that died showed severe bronchointerstitial pneumonia, including necrosis and sloughing of bronchial and bronchiolar epithelium; alveolar spaces filled with an exudate composed of foamy macrophages, neutrophils, and fibrin; and multifocal intraalveolar hemorrhage. In the second chimpanzee, we observed multifocal manifestations of bacilli-like bacteria. For the remaining 2 ill chimpanzees, clinical signs gradually disappeared in 6 days.

The keeper of the chimpanzees was a 31-year-old man, a nonsmoker who had a history of persistent allergic rhinoconjunctivitis and childhood asthma. He reported signs of a cold on June 21. His health deteriorated over the next 2 days; he had fever, sore throat, chills, muscle and joint pain, headache, and a dry cough. He visited the emergency department of Golnik University Clinic of Pulmonology and Allergic Diseases on June 23.

A nasopharyngeal swab specimen showed only hMPV by PCR. A throat swab specimen was negative for pathogenic bacteria. On July 1, the patient was hospitalized because of chest tightness and wheezing. Examination detected inspiratory and expiratory wheezing with crepitations. High-resolution computed tomography showed localized mucus plugs in the bronchi for right posterior basal segments with segmental air trapping and no radiologic signs of asthma or bronchiolitis. He was given inhaled methylprednisolone and high-dose salbutamol and was discharged on July 4.

To confirm that the respiratory infection had spread from chimpanzees to their keeper, we performed real-time reverse transcription PCR and sequenced PCR products of part of the hMPV fusion protein gene (*2*). At the time of the hMPV outbreak among the chimpanzees, hMPV was circulating in Slovenia. Among 39 samples available for June 3–July 2 from the population in central Slovenia, we sequenced 12 samples with the lowest cycle threshold values to facilitate insight into the molecular epidemiology of circulating hMPV variants.

Sequencing analyses showed that virus sequences from the chimpanzees and keeper had 100% identity (Figure), as did 2 other sequences from 2 patients hospitalized during the virus outbreak at the zoo. These 2 sequences showed 99% identity with a previously reported sequence (GenBank accession no. KC731511). Alignment of all 15 sequences and phylogenetic analyses showed 7 unique sequences (GenBank accession nos. MN978917–23) among the 12 selected samples.

**Figure:**
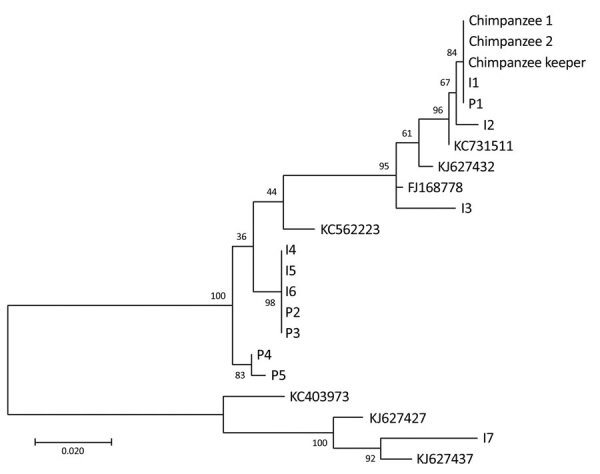
Phylogenetic tree of human metapneumovirus fusion protein gene fragments (518 bp) from chimpanzees and a keeper in a zoo, Slovenia, compared with randomly selected sequences from other patients with human metapneumovirus infections. Fusion protein gene fragments were inferred by using the maximum-likelihood method under a Tamura–Nei substitution model. I indicates sequences obtained from patients hospitalized at the Infectious Disease Department, University Medical Centre, Ljubljana, Slovenia; P indicates sequences obtained from patients hospitalized at the Pediatric Department at the same centre. Sequences I1–I7 are from samples collected during June 3–28, 2013, and sequences P1–P5 are from samples collected during June 4–July 2, 2013. All sequences were obtained from patients in the central region of Slovenia. Sequences with accession numbers were selected from GenBank. Numbers along branches are bootstrap values. Scale bar indicates nucleotide substitutions per site.

These cases are noteworthy because of how the infection spread. We assume that the chimpanzees acquired the virus from an infected child in a school or preschool group of children visiting the zoo as an end-of-year trip. The infection could have been spread by airborne route. Another possibility is that the chimpanzees acquired the virus from contaminated food of visitors; because of a narrow space (1.3 m) between visitors and the inner fence enclosing the chimpanzee cage, children could have thrown candies and other food onto the cage floor, and the chimpanzees might have eaten them.

Although the keeper had close contact with an ill chimpanzee and was probably infected by aerosols or body fluids from the chimpanzees, we cannot rule out the possibility that the keeper could have been infected by an infected adult human. We assume high infectivity pressure on the keeper, who fell ill only 2 days after the first chimpanzee started showing signs of respiratory infection. The keeper did not have children of his own and did not have close contact with children for at least 2 weeks before the incident.
